# Patient and heath system delays in the diagnosis and treatment of new and retreatment pulmonary tuberculosis cases in Malawi

**DOI:** 10.1186/1471-2334-14-132

**Published:** 2014-03-10

**Authors:** Lumbani Makwakwa, Mei-ling Sheu, Chen-Yuan Chiang, Shoei-Loong Lin, Peter W Chang

**Affiliations:** 1 School of Health Care Administration, Taipei Medical University, Taipei City, Taiwan; 2 Pharmaceutical Services Section, Christian Health Association of Malawi (CHAM), Lilongwe, Malawi; 3 Departments of Lung Health and NCDs, International Union Against Tuberculosis and Lung Disease, Paris, France; 4 Division of Pulmonary Medicine, Department of Internal Medicine, Wan Fang Hospital, Taipei Medical University, Taipei, Taiwan; 5 Department of Internal Medicine, College of Medicine, Taipei Medical University, Taipei, Taiwan; 6 Taipei Hospital, Ministry of Health and Welfare, New Taipei City, Taiwan; 7 College of Public Health and Nutrition, Taipei Medical University, Taipei, Taiwan

**Keywords:** Patient delay, Health system delay, Tuberculosis, Case detection

## Abstract

**Background:**

Tuberculosis (TB) control remains a challenge in Malawi despite the National TB Control Program since 1984. This study aimed at measuring patient and health system delays and identifying factors associated with these delays.

**Methods:**

A cross-sectional survey of 588 pulmonary TB patients was conducted in three TB centres in Blantyre, Lilongwe, and Mzuzu, between July and December 2011 using a semi-structured questionnaire. Patient delay was defined as the time interval between the onset of TB symptom(s) (a common symptom being coughing) to the first visit to any health provider. Health system delay was the interval from the first care-seeking visit at any health provider to the initiation of anti-tuberculosis treatment. Participants were invited to participate in the study during intensive phase of treatment. The characteristics associated with patient and health system delays were analyzed.

**Results:**

The median patient delay was 14 days for both new and retreatment TB cases (interquartile range [IQR] 14 – 28 and 7 – 21, respectively). The median health system delay was 59 days (IQR 26 – 108) for new and 40.5 days (IQR 21–90) for retreatment cases. Factors associated with longer patient delay in new cases included primary education (adjusted odds ratio [AOR] 2.2, 95% CI 1.3 – 3.9) and knowledge that more than three weeks of coughing is a sign of TB (AOR 1.9, 1.1 – 3.3). In retreatment cases, distance >10 Km (AOR 3.3, 1.1 – 9.6) and knowledge that more than three weeks of coughing is a sign of TB (AOR 3.7, 1.3 – 10.7; p < 0.05) were significant factors. Making the first visit to a health centre (OR 1.9, 0.9 – 3.8) or a drug store/ traditional healer (OR 5.1, 1.1 – 21.7) in new TB cases were associated with a longer health system delay (p < 0.05) while smear negative (OR 6.4, 1.5 – 28.3), and smear unknown or not done (OR 6.1, 1.3 – 26.9) among retreatment cases were associated with a longer health system delay (p < 0.05).

**Conclusions:**

Effective management and new diagnostic techniques are needed especially among retreatment cases. It is also needed to address geographic barriers to accessing care and increasing TB awareness in the community.

## Background

Tuberculosis (TB) remains a major health problem despite an increase in coverage of Directly Observed Treatment Short Course (DOTS) therapy and adoption of passive case detection globally. The goal of TB control is to reduce morbidity, mortality, transmission and development of drug resistance. TB control remains a major challenge in Malawi and the sub-Saharan region, which bears the biggest impact of HIV fuelled TB epidemics [1,2]. In Malawi, a total of 21, 092 TB cases were reported in 2010 with a notification rate of 142 per 100, 000 population. The estimated incidence of TB was 219 per 100, 000 population and the estimated case detection rate was 65%. The proportion of TB cases tested positive for HIV was 70% in 2010 [3].

Malawi had operated a vertical TB control program since 1984 and successfully implemented the DOTS strategy [4]. Smear microscopy was performed at referral and district hospitals and a few selected private and not-for-profit mission hospitals with microscopes. Treatment has been decentralized to rural health facilities [5]. During the study period, Malawi had an estimated population of 14,000,000 with a total of 213 microscopy centres and 88 initiation centres where patients could be diagnosed, registered and started on anti-tuberculosis treatment [4,6]. Sputum microscopy is the primary investigative method (gold standard) when suspecting TB in patients in Malawi.

Early case detection, prompt diagnosis and treatment of TB are essential for an effective TB control program. Despite services and medicines being given free of charge to TB patients and control strategies being in place for over two decades in Malawi, the estimated case detection rate of TB remained relatively low, likely indicating under-diagnosis as well as delays in the diagnosis and treatment of TB [3]. These may result in increased costs and disease severity in the individuals, as well as increased morbidity and mortality of TB in the community from increased risk of transmission of TB [7].

This study aims to measure the extent of and factors associated with patient delay in seeking health care, as well as health system delay in the diagnosis and treatment of new and retreatment TB cases in Malawi. An understanding of the main determinants of these delays is essential for formulating effective policies for TB control. Although studies on delays in TB diagnosis have been done before in Malawi [8,9], they only focused on new cases and none have been performed among new and retreatment TB cases simultaneously.

## Methods

### Study setting

A hospital based cross-sectional study was conducted at three main TB registration centres out of a total of 213 microscopy centres and 88 initiation centres where patients could be diagnosed, registered and started on anti-tuberculosis treatment between July and December 2011. A registration centre was chosen in Blantyre, Lilongwe and Mzuzu, representing the southern, central and northern regions of Malawi, respectively. These centres are located within the three major cities to provide services to a large number of TB cases, including referral cases. Passive case finding and sputum microscopy were the primary strategies used in the country during the study period.

### Study participants

The study population was composed of all registered pulmonary TB patients (new and retreatment cases), both male and female aged 15 years old or more, who were receiving the intensive phase of treatment at the facilities during the study period. In this study, new TB cases were patients who never had anti-TB treatment or who had taken anti-TB drugs for less than one month. Retreatment TB cases were patients who had previously been treated for TB for one month or more. The eligible patients who had consented to the interview were consecutively enrolled in the study during the time they were receiving the intensive phase of treatment at the facilities during the study period. Patients with chronic pulmonary TB, or those classified as treatment failure TB cases, or mentally or terminally ill were excluded.

### Data collection

Face-to-face interviews using a pretested questionnaire were employed to collect data in this study. Three nurses who had more than 5 years of experience in the management of TB patients under the National TB Program (NTP) in Malawi were trained to conduct the interview. Patients were asked to provide information concerning their demographic and socio-economic characteristics, knowledge of TB, duration of symptoms before seeking medical consultation and knowledge of the diagnosis and treatment process. The patient’s health passport, TB treatment card/ sheet and TB registers were evaluated where appropriate to verify the responses.

### Definition of delay

Patient delay was defined as the time interval between the onset of TB symptom(s) (a common symptom being coughing) to the first visit to any health provider. Health system delay was the interval from the first care-seeking visit at any health provider to the initiation of anti-tuberculosis treatment. Total delay was the time interval from the onset of symptoms to the initiation of anti-tuberculosis treatment.

### Data management and analysis

Data analysis was performed using IBM’s SPSS Version 19 for Microsoft Windows. The distributions of both delays were skewed and therefore non-parametric Kruskal Wallis one-way analysis of variance was employed to analyze associations between determinants and delays.

Further, the median patient delay of two weeks was used to dichotomize patient delay into acceptable (shorter) or longer delay. For the median health system delay a two week period was used to dichotomize shorter and longer health system delay as was recommended by Malawi NTP [4]. Multiple logistic regression analysis was used to analyze factors associated with longer patient and health system delays. *p*-values < 0.05 were considered statistically significant.

### Ethical consideration

Ethical permission to conduct the study was obtained from the National Health Sciences Research Committee (NHSRC) of Malawi. The objective of the study was explained in detail to study subjects and verbal consent was obtained from each individual before conducting the interview.

## Results

### Sociodemographic characteristics of pulmonary TB patients

A total of 588 pulmonary TB patients from three registration centres (Blantyre, Lilongwe and Mzuzu) receiving the intensive phase of TB treatment were consecutively enrolled in the study between July and December 2011. Table 1 shows the characteristics of pulmonary TB patients enrolled in this study. There were 460 (78.2%) new cases and 128 (21.8%) retreatment cases. After noticing symptoms, the most common health facility first visited by the patients was hospital (58.7%), followed by health centre (22.6%), drug store (8.8%), private clinic (7.7%), and traditional healer (2.2%). Of the 588 pulmonary TB patients, 389 (66.2%) were smear positive, 58 (9.9%) were smear negative, and 141 (24.0%) were smear result unknown or not done.

**Table 1:** Characteristics of pulmonary tuberculosis patients in Malawi, 2011

**Patient characteristics**	**N**	**(%)**
Total	588	(100.0)
TB case type		
New	460	(78.2)
Retreatment	128	(21.8)
Sex		
Male	388	(66.0)
Female	200	(34.0)
Age at diagnosis (years)		
15 – 24	86	(14.6)
25 – 34	211	(35.9)
35 – 44	161	(27.4)
45 – 54	69	(11.7)
55 – 64	40	(6.8)
65 +	21	(3.6)
Education		
No school	82	(13.9)
Primary	285	(48.5)
Secondary	186	(31.6)
Tertiary	35	(6.0)
Occupation		
Self-employed	201	(34.2)
Employed	132	(22.4)
Unemployed	85	(14.5)
Farmer	77	(13.1)
Housewife	63	(10.7)
Student	30	(5.1)
Average household monthly income (USD)		
≤ 30	105	(17.9)
> 30 ≤ 60	119	(20.2)
> 60	171	(29.1)
Unknown	193	(32.8)
Distance to the nearest TB diagnostic facility		
0 – 10 Km	364	(61.9)
11 – 20 Km	99	(16.8)
>20 Km	71	(12.1)
Unknown	54	(9.2)
Heard of TB before diagnosis		
Yes	396	(67.3)
No	192	(32.7)
Knowledge of coughing > 3 weeks as a TB symptom^a^		
Yes	268	(71.8)
No	105	(28.2)
Health facility first visited		
Hospital	345	(58.7)
Health centre	133	(22.6)
Drug store	52	(8.8)
Private clinic	45	(7.7)
Traditional healer	13	(2.2)
Sputum smear for acid-fast bacilli		
Negative	58	(9.9)
Positive	389	(66.2)
Unknown	141	(24.0)
Chest x-ray on diagnosis		
Yes	223	(37.9)
No	365	(62.1)

*Note:*^*a*^*n =373.*

### Patient delay and health system delay

The median patient delay was 14 days for both new and retreatment TB patients (interquartile range [IQR] 14 – 28 and 7 – 21, respectively). In new cases, occupation status (p = 0.017) was significantly associated with patient delay: the unemployed (median 21 days) and self-employed (median 17.5 days) had longer patient delays than those employed (median 14 days). Among patients who had heard of TB before diagnosis, knowledge that more than 3 weeks of coughing is a symptom of TB was significantly associated with patient delay in both new (p = 0.007) and retreatment cases (p = 0.018) (Tables 2 and 3).

**Table 2:** Patient and health system delay among new TB cases, by background characteristics, Malawi, 2011

		**Patient delay (days)**			**Health system delay (days)**		
	**n**	**Median**	**IQR**		**Median**	**IQR**	
Total	460	14.0	14-28		59.0	27-108	
Sex							
Male	304	14.0	14-28		59.0	28-99	
Female	156	21.0	14-30		64.0	24-125	
Age at diagnosis (years)							
< 34	221	21.0	14-30		59.0	27-100	
≥ 34	239	14.0	14-28		63.0	26-121	
Education							
No school	65	21.0	14-30		84.0	41-152	
Primary	219	21.0	14-28		57.0	27-107	
Secondary & above	176	14.0	7-28		54.0	24-91	
Occupation							
Unemployed	204	21.0	14-30	*	66.5	31-118	
Self-employed	146	17.5	14-30	*	51.5	24-92	
Employed	110	14.0	7-21	*	62.0	28-121	
Distance to nearest TB diagnostic facility							
0 – 10 Km	278	21.0	14-28		56.5	26-100	
>10 Km	140	21.0	14-28		73.5	32-119	
Unknown	42	14.0	13-28		47.5	21-133	
Heard of TB before diagnosis							
Yes	268	14.0	14-28		66.0	31-116	
No	192	21.0	14-28		50.5	22-98	
Knowledge of coughing > 3 weeks as a TB symptom							
Yes	186	21.0	14-30	*	73.0	29-116	
No	82	14.0	7-28	*	64.0	31-118	
Perceiving condition as worsening							
Yes	80	14.0	14-30		39.5	19-93	
No	380	17.5	14-28		63.0	31-115	
Health facility first visited							
Hospital	253	14.0	14-28		45.0	20-86	*
Health centre	114	21.0	14-30		86.0	43-135	*
Drug store& traditional	53	14.0	11-21		91.0	54-140	*
Private clinic	40	21.0	14-30		57.0	27-94	*
Smear							
Positive	302	14.0	14-28		62.5	28-111	
Negative	37	21.0	7-29		66.0	26-127	
Unknown/not done	121	21.0	14-29		56.0	25-98	

*Note: Using nonparametric Kruskal Wallis test*, IQR = interquartile range.

* p < 0.05.

**Table 3:** Patient delay and health system delay among retreatment TB cases, by background characteristics, Malawi, 2011

		**Patient delay (days)**			**Health system delay (days)**		
	**n**	**Median**	**IQR**		**Median**	**IQR**	
Total	128	14.0	7-21		40.5	22-91	
Sex							
Male	84	14.0	7-25		40.5	21-98	
Female	44	14.0	7-21		41.0	22-84	
Age at diagnosis (years)							
< 34	55	14.0	7-21		43.0	24-93	
≥ 34	73	14.0	7-28		39.0	21-88	
Education							
No school	17	14.0	14-28		62.0	21-95	
Primary	66	14.0	7-29		40.5	22-93	
Secondary & above	45	14.0	7-21		40.0	21-83	
Occupation							
Unemployed	51	14.0	7-28		43.0	27-82	
Self-employed	55	14.0	7-21		39.0	19-113	
Employed	22	14.0	7-21		39.5	19-104	
Distance to nearest TB diagnostic facility							
0 – 10 Km	86	14.0	7-21		47.5	23-95	
>10 Km	30	21.0	14-28		25.5	17-54	
Unknown	12	17.5	9-30		74.0	42-102	
Heard of TB before diagnosis							
Yes	128	14.0	7-21		40.5	22-91	
No	0	-	-		-	-	
Knowledge of coughing > 3 weeks as a TB symptom							
Yes	82	21.0	14-30	*	40.0	22-92	
No	23	14.0	7-28	*	41.5	20-107	
Perceiving condition as worsening							
Yes	23	14.0	7-28		41.0	19-73	
No	105	14.0	7-21		40.0	22-93	
Health facility first visited							
Hospital	92	14.0	7-21		38.0	22-71	*
Health centre	19	21.0	14-30		43.0	17-130	*
Drug store& traditional	12	14.0	14-28		123.5	85-189	*
Private clinic	5	7.0	7-14		76.0	18-133	*
Smear							
Positive	87	14.0	7-28		38.0	16-79	*
Negative	21	14.0	7-25		59.0	30-162	*
Unknown/ not done	20	14.0	7-20		68.5	28-97	*

*Note: Using nonparametric Kruskal Wallis test*, IQR = interquartile range.

* p < 0.05.

The proportion of new TB patients with patient delay longer than 2 weeks was 45% and that of retreatment cases was 32.8% (p = 0.015) while the proportion of new TB patients with health system delay longer than 2 weeks was 47.8% and that of retreatment cases was 50.8% (p = 0.031). Among new cases, primary education (adjusted odds ratio [AOR] 2.2, 95% CI 1.3 – 3.9) and knowledge that more than 3 weeks of coughing is a symptom of TB (AOR 1.9, 95% CI 1.1 – 3.3) were both significantly associated with longer patient delay (Table 4) when compared with other groups. For retreatment cases, knowledge that more than 3 weeks of coughing is a symptom of TB (AOR 3.7, 95% CI 1.3 – 10.7) and living at distances greater than 10 Kilometres (Km) from the nearest TB diagnostic facility (AOR 3.3, 95% CI 1.1 – 9.6) were significantly associated with longer patient delay when compared with other groups (Table 4). A cumulative 50.2% of new TB patients and 60.2% of retreatment cases had made visits to a health provider within 2 weeks after the onset of symptoms.

**Table 4:** Logistic regression analysis of determinants of longer patient delay, Malawi, 2011

**Characteristic**	**New TB cases**			**Retreatment TB cases**		
	**n**	**Unadjusted OR**	**Adjusted OR**	**n**	**Unadjusted OR**	**Adjusted OR**
		**(95% CI)**	**(95% CI)**		**(95% CI)**	**(95% CI)**
Total	460			128		
Education						
No school	65	1.47 (0.83 – 2.60)	1.73 (0.67 – 4.44)	17	1.27 (0.41 – 3.98)	
Primary	219	1.42 (0.95 – 2.11)	2.22 (1.25 - 3.94)*	66	1.34 (0.61 – 2.92)	
Secondary & above	176	reference		45	reference	
Occupation						
Unemployed	204	reference		51	reference	
Self-employed	146	0.86 (0.56 – 1.31)		55	0.95 (0.44 – 2.07)	
Employed	110	0.61 (0.39 – 0.98)		22	0.82 (0.29 – 2.29)	
Knowledge of coughing > 3 weeks as a TB symptom						
Yes	186	1.88 (1.11 – 3.19)	1.90 (1.10 –3.30)*	82	2.56 (0.99 – 6.61)	3.65 (1.25 –10.69)*
No	82	reference		23	reference	
Distance to nearest TB diagnostic facility						
0 – 10 Km	278	reference		86	reference	
> 10 Km	140	1.04 (0.69 – 1.57)		30	2.25 (0.97 – 5.23)	3.31 (1.14 – 9.59)*
Unknown	42	0.67 (0.35 – 1.30)		12	1.97 (0.58 – 6.64)	2.02 (0.38 – 10.76)
Perceiving condition as worsening						
Yes	80	0.95 (0.59 – 1.54)		23	0.96 (0.38 – 2.43)	
No	380	reference		105	reference	

OR = odds ratio, CI = confidence interval.

*p < 0.05.

The median health system delay was 59 days (IQR 26–108) for the new cases, which was longer than retreatment cases (40.5 days [IQR 21 – 90]) (p = 0.031). In new TB cases, the type of health facility first visited (p < 0.001) was significantly associated with health system delay (Table 2). For retreatment cases, the type of health facility first visited (p = 0.002) and smear results (p = 0.024) were significantly associated with health system delay (Table 3).

Further, using a period of 2 weeks to dichotomize health system delay into shorter and longer health system delay [10], making the first visit to a health centre (OR 1.9, 95% CI 0.9 – 3.8), or a drug store or a traditional healer (OR 5.1, 95% CI 1.1– 21.7) were significantly associated with longer health system delays among new TB cases as compared to visiting a hospital. For retreatment cases, smear negative (OR 6.4, 95% CI 1.5 – 28.3) and smear unknown or not done (OR 6.1, 95% CI 1.3 – 26.9) results were significantly associated with longer health system delay (Table 5). For health system delay, only a cumulative 14.5% of the patients had been started on treatment after two weeks from the first visit to health providers, including 13.5% for new cases and 18.0% fir retreatment cases.

**Table 5:** Logistic regression analysis of determinants of longer health system delay, Malawi, 2011

**Characteristic**	**New TB cases**		**Retreatment TB cases**	
	**n**	**Unadjusted OR (95% CI)**	**n**	**Unadjusted OR (95% CI)**
Total	460		128	
Place first visited by patients				
Hospital	253	reference	92	reference
Health centre	114	1.86 (1.92 – 3.77)*	19	0.91 (0.27 – 3.08)
Drug store & traditional	53	5.08 (1.08 -21.67)*	12	- ±
Private clinic	40	0.94 (0.39 – 2.26)	5	0.97 (0.10 – 9.24)
Smear				
Positive	302	reference	87	reference
Negative	37	0.65 (0.27 – 1.59)	21	6.36 (1.54 – 28.31)*
Unknown/not done	121	1.08 (0.57 – 2.04)	20	6.05 (1.26 – 26.91)*

OR = odds ratio, CI = confidence interval.

*p < 0.05.

### Total delay

The median total delay was 80 days (IQR 44–129). New cases had a longer median (85.5 days) total delay as compared with retreatment cases (60.5 days) (p = 0.002). The health system delay contributed more than 70% to the total delay. After a period of 4 weeks since the onset of symptoms only a cumulative 9.7% of the patients had been started on anti-tuberculosis treatment, with 9.1% for new cases and 11.7% for retreatment cases.

## Discussion

This study reveals that there were substantial delays between the onset of reported symptoms and the initiation of anti-tuberculosis treatment among pulmonary TB cases in Malawi with these delays mainly related to the health care system. The median health system delay in both new (59 days) and retreatment (40.5 days) TB cases are longer than patient delay (14 days) for both types of TB cases. A large proportion of patients (90%) had not been started on anti-tuberculosis treatment 4 weeks after the onset of presenting symptoms, and coughing being the most common symptom (89%) of these cases.

### Patient delay

The median patient delay (14 days) was found to be within the recommended target of 2 weeks and was shorter than median health system delays (59 days and 40.5 days for new and retreatment cases, respectively) [10]. Delays in the diagnosis and treatment of pulmonary TB have been shown to be associated with an increased risk of infectivity and poor clinical outcomes [11].

The median patient delay in this study for new cases was shorter than that found in earlier studies conducted in Malawi in 2000 (56 days) and 2008 (33.5 days); there were however no previous studies on retreatment cases for comparison [8,9]. The marked reduction in patient delay could be attributed to increased community awareness, increased access to diagnostic services and improved treatment outcomes following decentralization of TB services [4,12]. The median patient delay was also shorter than that in Ethiopia and Tanzania (30 days) [13,14], but longer than that in Taiwan (7 days) [15].

In this study, education was found to be significantly associated with patient delay in new cases, where those with only primary education had longer patient delay than those with secondary education. Not attending school and illiteracy were factors significantly associated with patient delay in studies in Gambia and seven countries of the Eastern Mediterranean Region [16,17]. However in this study, the no-school group was not significantly associated with patient delay. Nevertheless, patients with higher education likely had more opportunity to obtain information about TB.

In both new and retreatment cases, there was a significant association between patient delay and knowledge that more than 3 weeks of coughing is a symptom of TB. Those patients who knew that coughing for more than 3 weeks is a TB symptom were more likely to have longer delay. This is contradictory to common belief that knowledge of TB may trigger action of seeking health care. We therefore speculate that this may have happened either because the patients did not believe TB could happen to them, or they were waiting for the 3 week period to elapse before seeking care. They may also have feared being stigmatized, especially in Malawi where there is strong association between HIV/AIDS and TB in the face of high HIV/AIDS and TB co-infection rate (70%).

In retreatment cases, living at distances longer than 10 Km from a TB diagnostic facility was significantly associated with longer patient delays. This was in-line with the finding in the previous study in Malawi and therefore likely reveals barriers to accessing care among patients living in the suburbs and distant areas, thereby increasing the risk of transmission of TB [8]. An increase in TB diagnostic facilities and mobile clinics or sputum collection centres close to the communities might be an important step; that is, to decentralizing the services towards peripheral to enhance their diagnosis capacity and to reduce delays. Further innovative methods in case finding in the community should be considered to increase access to health services among those who have limited access to health care and close contacts to known TB patients [18].

### Health system delay

The alertness of the health system to the diagnosis and treatment of TB is essential to the control of TB in Malawi [19]. There were no previous studies done in Malawi that specified the health system delay period for comparison with the findings in this study. Nevertheless, the median health system delay (59 days for new cases and 40.5 days for retreatment cases) in this study was well beyond the recommended 2-weeks period for an effective health system to control TB [4,10]. As compared to the patient delay, the median health system delay was significantly longer and contributed a higher percentage (over 70%) to the total delay.

The type of health facility first visited by the patients was significantly associated with health system delay among new cases. Significant delays were especially among patients who made the first visits to a health centre and a drug store or a traditional healer. Long pathways to TB diagnosis including multiple patient visits to health centres and traditional healers, prescribing an antibiotic, and health system structural barriers were some of the findings causing delays to diagnosis of pulmonary TB in previous studies in Malawi [8,20-22]. This may have also resulted from hospitals generally having more qualified staff and more equipment, such as microscopes, than health centres. It is essential to sensitize drug stores and traditional healers and enhance the capacity of health centres to ensure prompt referral, diagnosis and treatment of TB.

During the study, sputum microscopy was the primary investigative method when suspecting TB as GeneXpert had not yet been piloted in the country. It was however noticed that in a quarter of the patients, results of sputum smears were not available at the initiation of treatment. We were not able to determine whether this was due to patients’ inability to produce sputum (dry cough), inconclusive smear microscopy results, or clinicians’ over reliance on chest X-rays. Nevertheless, subsequent smear examination results among these patients indicated that they were smear negative.

Similar to that among new TB patients, negative smear results and smear unknown or not done were significantly associated with longer health system delay among retreatment TB cases in this study. Significant delays between sputum examination and starting TB treatment among smear negative patients have been documented in an earlier study in Malawi, which ranged from 3 to 6 weeks [23]. The reasons for the delays in this previous study were assessing the outcomes of antibiotic treatment and arranging for a chest X-ray examination. Clinicians’ difficulties in making a diagnosis among smear negative TB patients who had submitted multiple sets of sputum samples and received more than two courses of antibiotics before starting TB treatment were noted in a previous study in Malawi [22]. Smear negative pulmonary TB therefore still remains a clinical problem in Malawi coupled with the high prevalence of HIV/TB co-infection and limited resources which may promote further disease progression and transmission [22,24,25]. Close monitoring of smear negative patients, effective management algorithms, and new (fast) diagnostic techniques tailored for a resource-poor setting to complement sputum microscopy are required to control delays among smear negative TB patients.

The study nevertheless had limitations. First, the study depended on the patient’s ability to recall their health-seeking period, which is subject to recall bias. The study did not measure the effect of TB and HIV/AIDS co-infection, which may have had major effects on the delays [26]. Lastly, this study was performed in the TB diagnostic facilities located in the cities where there is better access to services and therefore the extent of delays, and factors affecting the delays may be different from those in rural areas. Consequently this study provides estimates of delays in urban areas, which are considered to have better access to TB services and perhaps a higher risk of infection.

## Conclusion

This study reveals substantial delays in the diagnosis and treatment of pulmonary TB in Malawi, noting health care system delays being longer than patient delays. Smear negative results among retreatment cases were associated with longer health system delay. To reduce health system delays, effective management algorithms and new diagnostic techniques to complement sputum microscopy are required among smear negative patients including retreatment cases. In addition, there is need to sensitize drug stores and traditional healers for prompt referral. Peripheral diagnosis strategy should be employed in Malawi through expanding and enhancing the capacity of existing health centres. This would also address geographic barriers to accessing care complemented by increased TB awareness in the communities.

## Competing interests

The author(s) declare that they have no competing interests.

## Authors’ contributions

LM, MS, CC, and PC all contributed to the development and reviewing of the study protocol. LM and MS performed data collection, writing of manuscript, and analysis, and with equal contribution to the works. All five authors were involved in editing and revising the manuscript, and read and approved the final manuscript.

## Pre-publication history

The pre-publication history for this paper can be accessed here:

http://www.biomedcentral.com/1471-2334/14/132/prepub
